# Tumour-infiltrating lymphocytes: from prognosis to treatment selection

**DOI:** 10.1038/s41416-022-02119-4

**Published:** 2022-12-23

**Authors:** Koen Brummel, Anneke L. Eerkens, Marco de Bruyn, Hans W. Nijman

**Affiliations:** University of Groningen, University Medical Center Groningen, Department of Obstetrics and Gynecology, Groningen, The Netherlands

**Keywords:** Tumour immunology, Immunosuppression, Tumour biomarkers, Prognostic markers, Predictive markers

## Abstract

Tumour-infiltrating lymphocytes (TILs) are considered crucial in anti-tumour immunity. Accordingly, the presence of TILs contains prognostic and predictive value. In 2011, we performed a systematic review and meta-analysis on the prognostic value of TILs across cancer types. Since then, the advent of immune checkpoint blockade (ICB) has renewed interest in the analysis of TILs. In this review, we first describe how our understanding of the prognostic value of TIL has changed over the last decade. New insights on novel TIL subsets are discussed and give a broader view on the prognostic effect of TILs in cancer. Apart from prognostic value, evidence on the predictive significance of TILs in the immune therapy era are discussed, as well as new techniques, such as machine learning that strive to incorporate these predictive capacities within clinical trials.

## Introduction

The interaction between cancer and immune cells in the tumour microenvironment (TME) is thought to be crucial for the control of the development and progression of malignant tumours. Accordingly, tumour-infiltrating lymphocytes (TILs) have been identified in primary tumour tissue, tumour-bearing lymph nodes and metastases of numerous cancer types. TILs are defined as lymphocytes within and around cancer cells and have been associated with a survival benefit [1–5]. However, the prognostic role of TILs still remains controversial within different types of tumours. In the 2011 issue of the British Journal of Cancer, we performed a systematic review and meta-analysis on the prognostic value of subtypes of TILs in a variety of solid cancer types, including ovarian, colorectal, lung, hepatocellular, and renal cell cancer [4]. In this review, the prognostic significance of intratumoral CD3^+^, CD8^+^, and CD4^+^ T lymphocytes, and intratumoral FoxP3^+^ T regulatory T lymphocytes (Tregs) was assessed. In the systematic review, Gooden et al. identified a positive association of intratumoral CD3^+^ and CD8^+^ TILs on overall and progression-free survival [4]. In contrast, intratumoral CD4^+^ TILs were associated with a slightly improved overall survival (OS) and FoxP3^+^ regulatory TILs were not associated with overall survival [4]. Over the past decade, TILs have not only continued to be proven of prognostic value but evidence is also starting to emerge of their predictive significance in the immunotherapy era [6, 7]. Hence, in this review, we discuss the follow-up work of our original publication in 2011. In addition, we will give insight into the prognostic value of T-lymphocyte subsets beyond CD3^+^, CD8^+^ and CD4^+^ T lymphocytes and discuss how these data can be applied in clinical trials as well as a biomarker for immunotherapy.

### Search strategy

The Pubmed database was searched without limits to identify all relevant studies to the subject. Diverse search terms were used, including, but not limited to “tumour-infiltrating lymphocytes”, “TILs”, “T cells”, “T lymphocytes”, “CD3 T cells, “CD8 T cells”, “CD4 T cells”, “FoxP3 T cells”, “regulatory T cells”, “B cells”, “PD-1”, “Programmed Cell-death 1”, “CD103”, “CD39”, “TIGIT”, “TIM-3”, “LAG-3”, “CTLA-4” “Prognosis”, “Survival”, “Cancer”, “Outcome”, “Tertiary Lymphoid Structures”, “Machine-learning”, “Deep-learning”, “Immunohistochemistry”, “Immunotherapy”, “Checkpoint Inhibition”, “Prediction”, “Predictive Value”, “Biomarker”.

When available, studies were included starting from 2012, when not available older literature was used.

#### Prognostic value of ‘classical’ TILs

In 2011, we reported on a systematic review and meta-analysis of survival associated with the presence of CD3^+^ tumour-infiltrating lymphocytes (TILs). Briefly summarised, CD3^+^ TIL infiltration was associated with both progression-free survival (PFS) (HR 0.53; 95% CI 0.39–0.73) and overall survival (OS) (HR 0.58; 95% CI 0.43–0.78) across malignancies [4]. Since publication in 2011, the advent of high-dimensional cytometric, mass spectrometry, and RNA-based single-cell analysis of CD3^+^ TILs has significantly improved our understanding of this heterogenous T cell population, discussed in more detail below. Nevertheless, as a single marker for the prognostic benefit of T cells in tumours, high CD3^+^ TIL infiltration has proven highly reliable in follow-up studies and has been associated with an improved OS in almost all cancer types, including melanoma and breast, colorectal, gastric, hepatocellular, and head and neck cancer [8–15].

Apart from the frequently used parameters of presence and density of TILs, recent studies also show that the location of TILs and their spatial organization may carry additional prognostic value. Accordingly, a meta-analysis of Mei et al. showed that a high CD3^+^ TIL infiltrate in the invasive tumour margin had a positive correlation with OS (HR 0.63; 95% CI, 0.42–0.93) and disease-free survival (DFS) (HR 0.63; 95% CI, 0.35–0.68) in patients with colorectal cancer [11]. Conversely, a high CD3^+^ TIL infiltrate in the tumour centre or tumour stroma had no association with OS or DFS. Elomaa et al. recently made similar observations and found that high proximity and density scores of T lymphocytes were associated with better cancer-specific (CSS) and OS and they suggest that there are stronger survival associations based on the invasive margins as compared to the tumour centre [16]. Thus, CD3 has remained a reliable and consistent marker for the identification of tumour-infiltrating T cells with prognostic benefit across cancer types.

In addition to CD3^+^ T cells, our original work also made a crude distinction between the prognostic value of different T cell subsets. Perhaps the most obvious, although arguably least exciting, division in T cell subsets at the time was the classic differentiation of cytotoxic CD8 vs helper CD4 T cells. As described by Gooden and others, the presence of CD8^+^ TILs is associated with prognostic benefits for all tested survival endpoints (OS, DSS, PFS) [4] and across solid cancers. For CD8 in particular, large cohort studies have demonstrated robust prognostic benefit. For instance, a meta-analysis of 14 studies with 2015 patients in hepatocellular carcinoma found a positive correlation between high levels of CD8^+^ T lymphocytes and OS (HR 0.71; 95% CI 0.51–0.99; *P* = 0.04) and DFS (HR 0.66; 95% CI 0.47–0.92; *P* = 0.01) [17]. In ovarian cancer, a large cohort study of 1815 patients demonstrated that the presence of epithelial CD8^+^ T lymphocytes was concomitant with improved OS (HR 0.45; 95% CI 0.34–0.58; *P* = 0.001) and PFS (HR 0.46; 95% CI 0.25–0.67), independent of clinicopathological variables. In most studies, the presence of CD8^+^ TILs had a larger magnitude of effect than the presence of CD3^+^ TILs [18, 19]. However, even though CD8^+^ TILs are associated with prognostic benefits, the diverse functional profiles and markers of CD8^+^ TILs in the TME makes the interpretation complex [19]. Maibach et al. have proposed to label activation markers and/or effector molecules when it comes to the prognostic value of CD8^+^ TILs [3], a suggestion in-line with functional studies discriminating bystander from cancer cell-reactive CD8^+^ T cells.

In contrast to the above-described CD8^+^ counterparts, CD4^+^ TILs have received less overall attention in the field of tumour immunology. However, recent work on e.g., follicular helper cells and class I-negative tumours are steadily shifting this paradigm [20, 21]. In part, the difference in contribution may stem from the complexity in CD4^+^ T-cell differentiation. CD4^+^ T cells can broadly differentiate into immune-promoting T helper (Th) subsets, as well as the highly immune suppressive CD4^+^ regulatory cells (Tregs) with counteracting roles in anti-tumour immunity. Although their functions differ, we originally concluded that high CD4^+^ TILs were associated with a slightly improved OS in oesophageal, gastric, hepatocellular, renal cell and ovarian carcinoma. However, we did not show an association between high CD4^+^ TILs PFS and disease-specific survival (DSS). Yet, data on the prognostic value of CD4^+^ TILs in more recent meta-analysis is conflicting. In hepatocellular, gastric, and colorectal carcinoma, no significant correlation was found between high CD4^+^ TILs and OS [15, 17, 22]. Nonetheless, in most cancers, including melanoma and head and neck, ovarian, cervical, and cholangiocarcinoma, a positive association was reported between high CD4^+^ TILs and OS and/or PFS [8, 13, 14, 23–27]. As these results did not all distinguish different CD4 T cell subsets, it remains at present impossible to determine whether the prognostic value of CD4^+^ TILs is dependent on cancer type or heterogeneity in CD4 subsets, or both. Nevertheless, in studies where a (partial) discrimination was made, the CD4^+^ subtype, Th1, seemed to be a more consistent prognostic factor. For example, in breast cancer and primary melanoma, the expression of Th1-associated genes predicted an improved OS [3, 23]. Unfortunately, no recent meta-analyses reported the effect of CD4^+^ TILs on DSS in solid cancers.

The same conflicting reports on survival exist for FoxP3^+^ CD4 Tregs. In 2011, we concluded that Tregs did not have an impact on OS, DSS and PFS in most solid cancer, including ovarian, endometrial, cervix, breast, hepatocellular, renal cell, gastric and colorectal carcinoma. Studies from 2012 until 2022 report great discrepancy in the prognostic value of FoxP3^+^ TILs within variable tumour types [9, 11, 14, 15, 17, 28–36]. Contrary to the findings of Gooden et al., FoxP3^+^ TIL infiltration has been associated with poor prognosis for OS and relapse-free survival (RFS) in most cancers, such as breast, hepatocellular, gastric, ovarian, cervix, and cholangiocarcinoma [17, 26, 28, 29, 32, 35–37]. However, Asahi et al. [34] found no statistically significant correlation between high density intratumorally FoxP3^+^ TILs and OS in gastric cancer and cholangiocarcinoma. In addition, in melanoma FoxP3^+^ TILs were not a negative nor positive prognostic factor. Interestingly, in colorectal and head and neck cancer high levels of intratumorally FoxP3^+^ were associated with a good prognosis [14, 28, 29, 31]. Based on these diverse findings, biological properties within different microenvironments of specific tumour (sub)types seemed to play an important role. In fact, an hypoxic and acidic TME has been associated with the upregulation of chemokines that enhance recruitment of Tregs to the tumour and increase activity of these Tregs in the tumour, which highlights the importance of the TME [38–42]. Furthermore, Saito et al. [30] proposed that the discrepancies in prognostic value of FoxP3^+^ TILs could be attributed to different subtypes of FoxP3^+^ TILs, namely suppression-competent FoxP3^hi^ Tregs and non-suppressive FoxP3^low^ Tregs. Hence, attention is shifting towards analyses of subtypes of CD8^+^, CD4^+^ and FoxP3^+^ TILs instead of the population as a whole.

Finally, an analysis strategy that may generate more predictive data for survival than scoring individual TILs is to determine the ratios between T lymphocyte subsets. At the time of our original work, there were limited studies incorporating T lymphocyte ratios. As a consequence, we were only able to perform a pooled analysis on the CD8^+^/FoxP3^+^ ratio and concluded that a high CD8^+^/FoxP3^+^ ratio has a strong positive relation with OS. In the last decade, this association was validated in different studies in which a positive relation between a high CD8^+^/FoxP3^+^ ratio and better survival outcomes was observed [17, 43]. In addition, several studies have reported on the association between CD4^+^/FoxP3^+^ ratio and OS. Like the CD8^+^/FoxP3^+^ ratio, high CD4^+^/FoxP3^+^ ratios correlated with improved OS [17, 27, 33, 44].

#### Prognostic value of ‘novel’ TIL subsets

In 2011, our review primarily focused on the prognostic value of the classic subsets of TILs which was limited to CD3^+^, CD4^+^, CD8^+^ and ratios between these subtypes. However, in the last decade, substantial developments have taken place with respect to the identification and prognostic value of TILs beyond these classic T cell subsets, including subsets defined by markers CD103, CD39 and PD-1.

CD103, also known as integrin αEβ7 (*ITGAE*), is a heterodimeric transmembrane protein expressed primarily on epithelial-associated lymphocytes that is involved in cell adhesion, migration and lymphocyte homing of cells through binding to E-cadherin [45, 46]. Since E-cadherin is expressed in epithelial cells, CD103 TILs have been associated with immunity against cancers of epithelial origin. As a result, CD8^+^CD103^+^ TILS were strongly associated with increased OS, DSS and/or RFS in most cancer types, including urothelial cell carcinoma and ovarian, cervical, endometrial, breast, colorectal, gastric, and head and neck cancer [13, 47–61]. Importantly, the favourable prognostic value of CD103 was only found for intratumoral CD103^+^ TILs and for CD8^+^ TILs expressing CD103^+^. In fact, stromal CD103^+^ TILs were not associated with prolonged DFS and OS, and CD4^+^CD103^+^ TILs were associated with poor OS in gastric cancer [61, 62]. Contrary to other cancer types, CD8^+^CD103^+^ TIL frequencies in cutaneous squamous cell carcinoma were significantly associated with the development of metastasis and worse prognosis [63]. The same was found in clear cell renal cell carcinoma (ccRCC) in which high density of CD8^+^CD103^+^ predicted worse OS [64]. Interestingly, Duhen et al. [65] investigated the prognostic role of co-expression of CD39 and CD103 on CD8^+^ TILs and concluded that dual expression of both CD103 and CD39 was consistently better at predicting survival than CD103 alone. Altogether, these data show that CD103^+^ TILs have a positive prognostic value in most solid cancers and that co-expression of CD39 may improve the prognostic value of CD103 alone.

CD39, encoded by the gene *ENTPD1*, is an ectoenzyme that is responsible, together with CD73, for the generation of an immunosuppressive form of adenosine by converting adenosine triphosphate (ATP) into adenosine diphosphate (ADP) and cyclic adenosine monophosphate (cAMP) [66]. In the context of cancer, only limited and conflicting literature is available on the prognostic value of CD39^+^ TILs. In hepatocellular carcinoma (HCC) and rectal adenoma carcinoma, a higher frequency of CD8^+^CD39^+^ TILs was positively associated with improved OS [67, 68]. In contrast, CD39 expression was significantly associated with advanced tumour stage and worse survival rate in ccRCC, and bladder and small cell lung cancer [69–71]. Interestingly, in lung cancer and ccRCC, high expression of CD39 was correlated with abundance of immune suppressive factors, such as FOXP3^+^ and PD-1^+^ TILs [69, 72]. In fact, ccRCC patients who received immune checkpoint blockade (ICB) with high CD39 expression exhibited favourable OS compared to ccRCC patients with low CD39 expression [69].

Finally, the TIL marker that has received arguably the most attention in recent years is Programmed cell death protein 1 (PD-1). PD-1 is an inhibitor of both adaptive and innate immune responses and a marker of exhaustion in TILs displayed on the surface of both activated CD4^+^ and CD8^+^ T lymphocytes [73]. While several studies report on the prognostic effect of PD-1-ligand 1 (PD-L1), the prognostic effect of PD-1 expression on TILs is less frequently examined. Nevertheless, studies reporting on this topic present a varying view on the matter. Studies performed in patients with intrahepatic cholangiocarcinoma and nasopharyngeal carcinoma reported lower overall survival and a higher recurrence rate [27, 74], while studies performed in patients with HGSOC and NSCLC reported a positive correlation between the presence of PD-1^+^ TILs and disease-specific survival and OS respectively [75, 76]. This might suggest that the prognostic effect of PD-1 depends not only on its presence but also on tumour type. Interestingly, recent research by Thommen et al. looked into a transcriptionally and functionally distinct PD-1^+^ CD8^+^ T cell pool. They reported that high expression of PD-1 is also associated with overexpression of other inhibitory receptors such as TIM-3, LAG-3, TIGIT, 2B4 (CD244) and BTLA, where the first two were almost exclusively found on TILs with high PD-1 expression [77]. Even though upregulation of PD-1 has a detrimental effect on classic CD8^+^ T cell functions such as cytotoxic activity and cytokine production and secretion, the study shows that TILs with high expression of PD-1 also acquire novel effector functions. Specifically, the production and secretion of the effector chemokine *CXCL13*. As a single-receptor chemokine, CXCL13 binds only to CXCR5, which is expressed on B cells and certain types of CD4^+^ T cells. This suggests that the secretion of CXCL13 by PD-1^+^ TILs attracts B cells and CD4 cells to the TME. This is substantiated by the fact that most of the CD8^+^ T cells with high PD-1 expression are colocalized with—among others—B cells in tertiary lymphoid structures (TLS). This new acquired effector function of exhausted PD-1 expressing T cells make them a possible predictor for response to PD-1 targeting therapies [77, 78]. Finally, although not studied extensively in meta-analyses, the presence of exhaustion markers TIGIT, TIM-3, LAG-3, CTLA-4 alone have been alternately correlated with OS and recurrence rate in isolated studies [79–83].

#### The prognostic value of TIL heterogeneity

Most studies evaluating the prognostic value of ‘classical’ and ‘novel’ TILs in solid tumours have been limited to the assessment of individual TIL markers by immunohistochemistry or mRNA expression. As density-based analyses of TILs are performed on biopsy slides that capture only a small part of the tumour, these assessments do not entirely explore the characteristics (and therefore prognosis) of tumours, including the spatial heterogeneity of TILs in the TME [84, 85]. In addition, immunohistochemistry on H&E slides is interpreted by pathologists, resulting in a highly subjective prognostic tool with a restricted reproducibility [86]. Recently, new technological breakthroughs in pathology and radiochemistry, such as machine learning and immune-positron emission tomography (immune-PET), have been developed that hold great promise in refining the prognostic value of TILs.

In 2012, Krizhevsky et al. [87] introduced convolutional neural networks, followed by the appearance of machine-learning (ML) models in pathology. In immune-oncology, ML, and in particularly deep learning (DL), has proven an unbiased and reproducible tool to identify histopathological patterns based on fully automated computer-aided image analysis of routinely generated H&E-stained slides. This is done by a.o. assessing consistency in the expression of immunohistochemical markers, tumour morphology, molecular alterations, and spatial distribution of TILs and cancer cells [85]. ML models have already been trained in a variety of cancers [84, 88–97].

Image-based DL algorithm to quantify TIL densities and to assess spatial heterogeneity of TILs might therefore augment the prognostic value of TILs significantly [88–90]. Heindl et al. [88] developed an image-based DL tool to score lymphocytic infiltration based on spatial heterogeneity of TILs in breast cancers and concluded that these scores were highly prognostic, particularly for late recurrences. Moreover, Horeweg et al. [89] confirmed that the integration of image-based quantification of intraepithelial CD8^+^ cells superseded the prognostic utility of the standard molecular endometrial cancer classification in early-stage endometrial cancer. Accordingly, prognostic image-based DL models have the advantage that they can potentially take into account the spatial interaction among TILs and cancer cells which has proven to have a predictive value in tumour progression and recurrence.

Altogether, these studies show that the prognostic value of TILs in clinical practice could be further enhanced by the utilisation of ML models.

#### TILs as a biomarker for immunotherapy

Blocking the above-mentioned PD-1-PD-L1 axis with monoclonal antibodies (MAbs) has increased therapeutic options in solid tumours. However, a significant group of patients do not benefit from PD-1/PD-L1 blockade, but are exposed to (the risk of) treatment-related toxicity. Because of this, there is a clinical need for prognostic and predictive biomarkers that can help reduce possible overtreatment. A logical biomarker of interest are TILs. A recently performed systematic review by Presti et al. gives a thorough overview of current research on TILs as a predictive biomarker [98]. A high baseline TIL density is associated with improved outcome (ORR in metastatic and pathological complete responses (pCR) in early disease) in several solid tumours treated with immune checkpoint inhibitors including melanoma, breast cancer, endometrial cancer, CRC, and NSCLC [7, 28, 99–111]. Furthermore, trials that evaluated on-treatment histological samples showed that the dynamics in TIL density during treatment was associated with improved outcome, even when there was no association with baseline TILs observed [100, 112]. Next to the density and variation of TILs in the TME, spatial distribution also influences the response to ICB. The ratio between the invasive margin and the tumour centre might be of special interest as it has been shown to provide additional information on early changes after administration of ICB in and around the tumour and might be an early predictive biomarker for treatment effects [101, 102, 107, 113–115]. Findings like these show that the TME is a dynamic system that constantly levitates on changes in the tumour-host interaction and that the dynamics in TILs, especially during and after treatment with ICB, can be of prognostic and predictive value in patients treated with ICB.

The importance of the TME on TIL dynamics is further proven by the effect of hypoxia and acidity on TIL effector functions and proliferation. It has been suggested that an acidic environment prevents lymphocyte proliferation by impairing the stimulatory activity of IL-2 [76, 116, 117]. Furthermore, acidosis in the TME impairs the cytolytic activity and cytokine secretion of CD8^+^ T lymphocytes [76, 118]. Hypoxia, often due to chaotic and insufficient tumour microcirculation [119], has detrimental effects on effector functions of both CD4^+^ and CD8^+^ T lymphocytes and supports the proliferation and migration of immunosuppressive cells. Furthermore, Zandberg et al. showed that the effect of anti-PD-1 therapy is diminished with increased hypoxia in HNSCC [120].

Apart from focusing on the predictive and productive value of TILs as a whole, alternative biomarkers such as TIL subsets (e.g., CD4^+^, Tregs, T-memory, CD8^+^), the expression of exhaustion (e.g., PD-1, TIM-3, LAG-3) and activation (e.g., granzyme B) markers and their association with clinical outcome after ICB treatment [108, 109] have also been studied. Even though these studies do not show a definitive function of one of these markers as a predictive biomarker, it shows the interaction between TILs and the TME in response to ICB treatment. Furthermore, the predictive value of a certain TIL can vary depending on the type of ICB that is used [109]. Even though these data on TILs in tumours show a possible predictive role of TILs in association with ICB treatment, a large number of studies lack an ICB-free arm for comparison complicating definitive conclusions. Finally, a high infiltration of TILs and the presence or absence of exhaustion and/or activation markers is not always correlated to a good response or any response at all. An example of this is ovarian cancer where several clinical trials with immune therapy show little-to-no response to ICB, even in tumours with high densities of TILs [121, 122].

DL may also play a crucial role in the selection of patients for immunotherapy. Indeed, Saltz et al. [84] used image-based DL to cluster spatially connected regions of TILs and found differences in cluster dispersion between melanoma, a cancer type that is highly response to immunotherapy, and breast cancer, a cancer type that is generally unresponsive to immunotherapy. Likewise, Chen et al. [91] identified and validated three distinct immune subtypes presented with diverse components of tumour-infiltrating immune cells, molecular features, and clinical characteristics in gastric cancer by using unsupervised consensus clustering algorithm. Thus, each cancer subtype may benefit from different immunotherapy strategies. Next to image-based DL, immune methylome signatures queried by ML were also shown to be predictive for immunotherapy response. Duruisseaux et al. found a correlation between epigenetic features based on DNA methylation signature (EPIMMUNE) and clinical benefit with PD-1 blockade in NSCLC. NSCLC tumours of non-responders to immunotherapy were enriched with cell populations derived from the myeloid lineage, while responders were enriched with cell populations derived from the lymphoid lineage [92]. Based on these studies (mixed), ML models may be a valuable tool to select patients for immunotherapy.

Image-based (TIL) DL has also gained attention for predicting the status of molecular pathways, including microsatellite instability (MSI) and mismatch repair deficiency (MMRd) status. In colorectal cancer, variable image-based DL models have been designed that exceeded the performance of experienced gastrointestinal pathologist at predicting MSI on H&E-stained slides [93–95]. Within these models, the presence of TILs and their spatial orientation in the tumour had important predictive value [94–97]. For instance, Lee et al. [96] confirmed that their image-based DL-model discriminated MSI-high and microsatellite stability (MSS) largely based on high TIL and peritumoral lymphocytosis. In addition, Schrammen et al. [94], Bilal et al. [95] and Kather et al. [97] found that lymphocyte-rich tumour regions, high proportions of TILs and necrotic tumour cells were most predictive for MSI in their image-based DL models. Since, MSI/MMRd status of a patient has therapeutic consequence, a cost-efficient image-based DL model using, e.g., TILs may prove to be an efficient tool to triage patients for confirmatory MSI/MMRd testing.

#### Perspectives

It is evident that TILs are associated with improved long-term survival across malignancies. A major challenge is to now translate these associations into clinically relevant or clinically actionable information. Indeed, while TIL ’scores’ may help supplement molecular information and improve patient counselling on the likelihood of recurrence, validated systems that can be implemented into routine clinical practice are scarce. This is true for both standardised scoring systems for pathologists, but also machine-learning algorithms. Arguably the most substantial obstacle that has hampered this clinical translation is the heterogeneity in spatiotemporal distribution of immune cells, and the underlying intra-immune cell heterogeneity. These problems are compounded by the apparent incongruity between the prognostic value of TILs in malignancies, and the potential likelihood of response of a malignancy to TIL-targeted immunotherapy, such as immune checkpoint inhibitors. A prototypical example of this paradox, ovarian cancer, has long been known to harbour tumour-reactive TILs with prognostic value, but only marginal responses to immune checkpoint inhibitors have been observed so far. Furthermore, as the prognostic value of TIL infiltrate in ovarian cancer appears to be restricted to a subgroup of primary patients with complete cytoreduction, it will be interesting to determine whether this subset also responds to ex vivo immune checkpoint inhibitor treatment in e.g., patient-derived tissue fragment (PDTF) models, or whether the prognostic and predictive value of TILs are uncoupled in these patients. As recent work points to tissue-resident memory-driven immune responses in ovarian cancer, and the therapeutic benefit of ICB is more and more linked to the involvement of (secondary) lymphoid organs, a more complex view on prognostic tissue-resident and predictive systemic immune responses may develop in the coming years.

An exciting development herein, both from patient and translational perspective, is the advent of neo-adjuvant ICB. Most successfully applied in MMRd CRC, neo-adjuvant ICB is associated with remarkable rates of pathologic complete responses. While a general trend is observed in these studies for higher levels of baseline CD8^+^ TILs in responding patients, the same trend does not hold for CD3^+^ TILs, and responses on the individual patient level are more heterogenous. With studies using radiolabeled immune imaging agents (e.g., CD8 and PD-1) now underway to tackle the problem of sampling heterogeneity, it will be interesting to see these modalities applied within the neo-adjuvant setting. Ideally, these whole-body immune monitoring agents will be applied in combination with an in-depth assessment of TLS and tumour-draining lymph nodes (TDLNs). Both TLS and TDLNs have been proposed as reservoirs for precursor-exhausted T cells that maintain long-term immunity against chronic stimulation by cancer cells, and have been linked to response to ICB, in many instances with superior predictive power to TIL status. However, major outstanding questions on the relationship between TILs, TLS and TDLNs remain to be addressed, most notably whether T-/B cell clones are shared across these sites, whether unique phenotypes exist across these sites, and how these cells dynamically respond to treatment with ICB. Technological developments on machine learning combined with high-dimensional techniques such as imaging mass cytometry and spatial transcriptomics are now starting to shed light on these questions, and it will be exciting to see this field develop over the coming years.

Overall, the analysis of the prognostic value of TILs combined with the clinical success of ICB-therapy has sparked an amazing development in our understanding of local and systemic tumour/immune cell interactions. Ever advancing high-dimensional assessment of immune cell control of tumours will need to be paired with an effective practical translation of this information into understandable, relevant and actionable information for use in clinical practice.

## Data Availability

Not applicable.

## References

[1] Fridman WH, Pagès F, Saut̀s-Fridman C, Galon J (2012). The immune contexture in human tumours: impact on clinical outcome. Nat Rev Cancer..

[2] Zhang L, Conejo-Garcia JR, Gimotty PA, Massobrio M, Regnani G, Makrigiannakis A, et al. Intratumoral T cells, recurrence, and survival in epithelial ovarian cancer. N Engl J Med. 2003;348:203–13. 10.1056/NEJMoa020177 12529460

[3] Maibach F, Sadozai H, Seyed Jafari SM, Hunger RE, Schenk M (2020). Tumor-infiltrating lymphocytes and their prognostic value in cutaneous melanoma. Front Immunol.

[4] Gooden MJM, De Bock GH, Leffers N, Daemen T, Nijman HW (2011). The prognostic influence of tumour-infiltrating lymphocytes in cancer: a systematic review with meta-analysis. Br J Cancer.

[5] Galon J, Costes A, Sanchez-Cabo F, Kirilovsky A, Mlecnik B, Lagorce-Pagès C (2006). Type, density, and location of immune cells within human colorectal tumors predict clinical outcome. Science.

[6] Badalamenti G, Fanale D, Incorvaia L, Barraco N, Listì A, Maragliano R, et al. Role of tumor-infiltrating lymphocytes in patients with solid tumors: can a drop dig a stone? Cell Immunol. 2019;343:103753. 10.1016/j.cellimm.2018.01.013 29395859

[7] Loi S, Michiels S, Adams S, Loibl S, Budczies J, Denkert C (2021). The journey of tumor-infiltrating lymphocytes as a biomarker in breast cancer: clinical utility in an era of checkpoint inhibition. Ann Oncol.

[8] Fu Q, Chen N, Ge C, Li R, Li Z, Zeng B, et al. Prognostic value of tumor-infiltrating lymphocytes in melanoma: a systematic review and meta-analysis. Oncoimmunology. 2019;8:e1593806. 10.1080/2162402X.2019.1593806 PMC6527267 31143514

[9] Erdag G, Schaefer JT, Smolkin ME, Deacon DH, Shea SM, Dengel LT, et al. Microenvironment and immunology immunotype and immunohistologic characteristics of tumor-infiltrating immune cells are associated with clinical outcome in metastatic melanoma. Cancer Res. 2012;72:1070–80. 10.1158/0008-5472.CAN-11-3218 PMC3306813 22266112

[10] Rathore AS, Kumar S, Konwar R, Srivastava AN, Makker A, Goel MM (2013). Presence of CD3^+^ tumor infiltrating lymphocytes is significantly associated with good prognosis in infiltrating ductal carcinoma of breast. Indian J Cancer.

[11] Mei Z, Liu Y, Liu C, Cui A, Liang Z, Wang G (2014). Tumour-infiltrating inflammation and prognosis in colorectal cancer: systematic review and meta-analysis. Br J Cancer.

[12] Arigami T, Uenosono Y, Ishigami S, Matsushita D, Hirahara T, Yanagita S, et al. Decreased density of CD3^+^ tumor-infiltrating lymphocytes during gastric cancer progression. J Gastroenterol Hepatol. 2014;29:1435–41. 10.1111/jgh.12551 25587614

[13] Hao J, Yu H, Zhang T, An R, Xue Y (2020). Prognostic impact of tumor-infiltrating lymphocytes in high grade serous ovarian cancer: a systematic review and meta-analysis. Ther Adv Vaccines.

[14] de Ruiter EJ, Ooft ML, Devriese LA, Willems SM. The prognostic role of tumor infiltrating T-lymphocytes in squamous cell carcinoma of the head and neck: a systematic review and meta-analysis. Oncoimmunology. 2017;6:e1356148. 10.1080/2162402X.2017.1356148 PMC5674970 29147608

[15] Yu PC, Long D, Liao CC, Zhang S. Association between density of tumor-infiltrating lymphocytes and prognoses of patients with gastric cancer. Medicine (Baltimore). 2018;97:e11387. 10.1097/MD.0000000000011387 PMC6076141 29979429

[16] Elomaa H, Ahtiainen M, Väyrynen SA, Ogino S, Nowak JA, Friman M, et al. Prognostic significance of spatial and density analysis of T lymphocytes in colorectal cancer. Br J Cancer. 2022;127:514–23. 10.1038/s41416-022-01822-6 PMC9345858 35449453

[17] Yao W, He JC, Yang Y, Wang JM, Qian YW, Yang T, et al. The prognostic value of tumor-infiltrating lymphocytes in hepatocellular carcinoma: a systematic review and meta-analysis. Sci Rep. 2017;7:1–11. 10.1038/s41598-017-08128-1 PMC5548736 28790445

[18] Hwang WT, Adams SF, Tahirovic E, Hagemann IS, Coukos G (2012). Prognostic significance of tumor-infiltrating T cells in ovarian cancer: a meta-analysis. Gynecol Oncol.

[19] Barnes TA, Amir E (2017). HYPE or HOPE: the prognostic value of infiltrating immune cells in cancer. Br J Cancer.

[20] Eschweiler S, Clarke J, Ramírez-Suástegui C, Panwar B, Madrigal A, Chee SJ (2021). Intratumoral follicular regulatory T cells curtail anti-PD-1 treatment efficacy. Nat Immunol.

[21] Vries NL de, Haar J van de, Veninga V, Chalabi M, Ijsselsteijn ME, Ploeg M van der, et al. γδ T cells are effectors of immune checkpoint blockade in mismatch repair-deficient colon cancers with antigen presentation defects. bioRxiv:2021.10.14.464229 [Preprint] 2021. [cited 2022 Sep 19]. Available from: https://www.biorxiv.org/content/10.1101/2021.10.14.464229v1

[22] Toor SM, Murshed K, Al-Dhaheri M, Khawar M, Abu Nada M, Elkord E (2019). Immune checkpoints in circulating and tumor-infiltrating CD4^+^ T cell subsets in colorectal cancer patients. Front Immunol.

[23] Stanton SE, Disis ML. Clinical significance of tumor-infiltrating lymphocytes in breast cancer. J ImmunoTherapy Cancer. 2016;4:1–7. 10.1186/s40425-016-0165-6 PMC5067916 27777769

[24] Borsetto D, Tomasoni M, Payne K, Polesel J, Deganello A, Bossi P (2021). Prognostic significance of cd4+ and cd8+ tumor-infiltrating lymphocytes in head and neck squamous cell carcinoma: a meta-analysis. Cancers.

[25] Rodrigo JP, Sánchez-Canteli M, López F, Wolf GT, Hernández-Prera JC, Williams MD, et al. Tumor-infiltrating lymphocytes in the tumor microenvironment of laryngeal squamous cell carcinoma: systematic review and meta-analysis. Biomedicines. 2021;9:486. 10.3390/biomedicines9050486 PMC8145951 33925205

[26] Liu D, Heij LR, Czigany Z, Dahl E, Lang SA, Ulmer TF (2022). The role of tumor-infiltrating lymphocytes in cholangiocarcinoma. J Exp Clin Cancer Res.

[27] Tang Y, Zhang AXJ, Chen G, Wu Y, Gu W (2021). Prognostic and therapeutic TILs of cervical cancer—current advances and future perspectives. Mol Ther - Oncolytics.

[28] Huang Y, Liao H, Zhang Y, Yuan R, Wang F (2014). Prognostic value of tumor-infiltrating FoxP3 + T cells in gastrointestinal cancers: a meta analysis. PLoS ONE.

[29] DeLeeuw RJ, Kost SE, Kakal JA, Nelson BH (2012). The prognostic value of FoxP3+ tumor-infiltrating lymphocytes in cancer: a critical review of the literature. Clin Cancer Res.

[30] Saito T, Nishikawa H, Wada H, Nagano Y, Sugiyama D, Atarashi K, et al. Two FOXP3+CD4+ T cell subpopulations distinctly control the prognosis of colorectal cancers. Nat Med. 2016;22:679–84. 10.1038/nm.4086 27111280

[31] Hu G, Li Z, Wang S (2017). Tumor-infiltrating FoxP3+ Tregs predict favorable outcome in colorectal cancer patients: a meta-analysis. Oncotarget..

[32] Shou J, Zhang Z, Lai Y, Chen Z, Huang J Worse outcome in breast cancer with higher tumor-infiltrating FOXP3+ Tregs: a systematic review and meta-analysis. BMC Cancer. 2016;16:1–8. 10.1186/s12885-016-2732-0 PMC5002190 27566250

[33] West NR, Kost SE, Martin SD, Milne K, Deleeuw RJ, Nelson BH, et al. Tumour-infiltrating FOXP3+ lymphocytes are associated with cytotoxic immune responses and good clinical outcome in oestrogen receptor-negative breast cancer. Br J Cancer. 2013;108:155–62. 10.1038/bjc.2012.524 PMC3553524 23169287

[34] Asahi Y, Kanako, Hatanaka C, Hatanaka Y, Kamiyama T, Orimo T (2020). Prognostic impact of CD8^+^ T cell distribution and its association with the HLA class I expression in intrahepatic cholangiocarcinoma. Surg Today.

[35] Vigano L, Soldani C, Franceschini B, Cimino M, Lleo A, Donadon M, et al. Tumor-infiltrating lymphocytes and macrophages in intrahepatic cholangiocellular carcinoma. Impact on Prognosis after Complete Surgery. J Gastrointest Surg. 2019;23:2216–24. 10.1007/s11605-019-04111-5 30843133

[36] Cao M, Wang Y, Wang D, Duan Y, Hong W, Zhang N (2020). Increased high-risk human papillomavirus viral load is associated with immunosuppressed microenvironment and predicts a worse long-term survival in cervical cancer patients. Am J Clin Pathol.

[37] Maeda N, Yoshimura K, Yamamoto S, Kuramasu A, Inoue M, Suzuki N (2014). Expression of B7-H3, a potential factor of tumor immune evasion in combination with the number of regulatory T cells, affects against recurrence-free survival in breast cancer patients. Oncology..

[38] Rao D, Verburg F, Renner K, Peeper DS, Lacroix R, Blank CU. Metabolic profiles of regulatory T cells in the tumour microenvironment. Cancer Immunol Immunother. 2021;70:2417–27. 10.1007/s00262-021-02881-z PMC10991195 33576875

[39] Mortezaee K, Majidpoor J. The impact of hypoxia on immune state in cancer. Life Sci. 2021;286:120057. 10.1016/j.lfs.2021.120057 34662552

[40] Huber V, Camisaschi C, Berzi A, Ferro S, Lugini L, Triulzi T, et al. Cancer acidity: an ultimate frontier of tumor immune escape and a novel target of immunomodulation. Semin Cancer Biol. 2017;43:74–89. 10.1016/j.semcancer.2017.03.001 28267587

[41] Watson MLJ, Vignali PDA, Mullett SJ, Overacre-Delgoffe AE, Peralta RM, Grebinoski S (2021). Metabolic support of tumour-infiltrating regulatory T cells by lactic acid. Nature..

[42] Suthen S, Lim CJ, Nguyen PHD, Dutertre CA, Lai HLH, Wasser M (2022). Hypoxia-driven immunosuppression by Treg and type-2 conventional dendritic cells in HCC. Hepatology..

[43] Tavares MC, Sampaio CD, Lima GE, Andrade VP, Gonçalves DG, Macedo MP, et al. A high CD8 to FOXP3 ratio in the tumor stroma and expression of PTEN in tumor cells are associated with improved survival in non-metastatic triple-negative breast carcinoma. BMC Cancer. 2021;21:1–12. 10.1186/s12885-021-08636-4 PMC8343973 34362334

[44] Fluxá P, Rojas-Sepúlveda D, Gleisner MA, Tittarelli A, Villegas P, Tapia L, et al. High CD8^+^ and absence of Foxp3^+^ T lymphocytes infiltration in gallbladder tumors correlate with prolonged patients survival. BMC Cancer. 2018;18:243. 10.1186/s12885-018-4147-6 PMC5833069 29499656

[45] Hoffmann JC, Schön MP, Leggatt G, Libertad Gonzalez Cruz J. Integrin αE (CD103)β 7 in epithelial cancer. Cancers. 2021;13:6211. 10.3390/cancers13246211 PMC8699740 34944831

[46] Kim Y, Shin Y, Gyeong &, Kang H Prognostic significance of CD103+ immune cells in solid tumor: a systemic review and meta-analysis. Sci Rep. 2019;9:1–7. 10.1038/s41598-019-40527-4 PMC6405906 30846807

[47] Webb JR, Milne K, Watson P, DeLeeuw RJ, Nelson BH (2014). Tumor-infiltrating lymphocytes expressing the tissue resident memory marker cd103 are associated with increased survival in high-grade serous ovarian cancer. Clin Cancer Res.

[48] Bösmüller HC, Wagner P, Peper JK, Schuster H, Pham DL, Greif K, et al. Combined immunoscore of CD103 and CD3 identifies long-term survivors in high-grade serous ovarian cancer. Int J Gynecological Cancer. 2016;26:671–9. 10.1097/IGC.0000000000000672 26905331

[49] Workel HH, Komdeur FL, Wouters MCA, Plat A, Klip HG, Eggink FA (2016). CD103 defines intraepithelial CD8^+^ PD1+ tumour-infiltrating lymphocytes of prognostic significance in endometrial adenocarcinoma. Eur J Cancer.

[50] Wang B, Wu S, Zeng H, Liu Z, Dong W, He W, et al. CD103 D tumor infiltrating lymphocytes predict a favorable prognosis in urothelial cell carcinoma of the bladder. J Immunol. 2015;194:556–62. 10.1016/j.juro.2015.02.2941 25752441

[51] Mann JE, Smith JD, Birkeland AC, Bellile E, Swiecicki P, Mierzwa M (2019). Analysis of tumor-infiltrating CD103 resident memory T-cell content in recurrent laryngeal squamous cell carcinoma Laryngeal squamous cell carcinoma OS overall survival PD-1 programmed cell death protein 1 RT radiation TIL tumor-infiltrating lymphocyte(s). Cancer Immunol, Immunother.

[52] Chu Y, Liao J, Li J, Wang Y, Yu X, Wang J (2019). CD103^+^ tumor-infiltrating lymphocytes predict favorable prognosis in patients with esophageal squamous cell carcinoma. J Cancer.

[53] Li R, Liu H, Cao Y, Wang J, Chen Y, Qi Y (2020). Identification and validation of an immunogenic subtype of gastric cancer with abundant intratumoural CD103^+^CD8^+^ T cells conferring favourable prognosis. Br J Cancer.

[54] Wang ZQ, Milne K, Derocher H, Webb JR, Nelson BH, Watson PH (2016). CD103 and intratumoral immune response in breast cancer. Clin Cancer Res.

[55] Park MH, Kwon SY, Choi JE, Gong G, Bae YK, PM H (2020). Intratumoral CD103-positive tumour-infiltrating lymphocytes are associated with favourable prognosis in patients with triple-negative breast cancer Intratumoral CD103-positive tumour-infiltrating lymphocytes are associated with favour-able prognosis in patients with triple-negative breast cancer. Histopathology..

[56] Hu W, Sun R, Chen L, Zheng X, Jiang J (2019). Prognostic significance of resident CD103^+^CD8^+^T cells in human colorectal cancer tissues. Acta Histochem.

[57] Komdeur FL, Prins TM, Van De Wall S, Plat A, Bea G, Wisman A, et al. CD103^+^ tumor-infiltrating lymphocytes are tumor-reactive intraepithelial CD8^+^ T cells associated with prognostic benefit and therapy response in cervical cancer View supplementary material CD103C tumor-infiltrating lymphocytes are tumor-reactive intraepithelial CD8C T cells associated with prognostic benefit and therapy response in cervical cancer. Oncoimmunol. 2017;6:e1338230. 10.1080/2162402X.2017.1338230 PMC5599086 28932636

[58] Edwards J, Wilmott JS, Madore J, Gide TN, Quek C, Tasker A (2018). CD103^+^ tumor-resident CD8^+^ T cells are associated with improved survival in immunotherapy-naïve melanoma patients and expand significantly during anti-PD-1 treatment. Clin Cancer Res.

[59] Djenidi F, Adam J, Goubar A, Durgeau A, Meurice G, de Montpréville V (2015). CD8^+^ CD103^+^ tumor-infiltrating lymphocytes are tumor-specific tissue-resident memory T cells and a prognostic factor for survival in lung cancer patients. J Immunol.

[60] Ganesan AP, Clarke J, Wood O, Garrido-Martin EM, Chee SJ, Mellows T (2017). Tissue-resident memory features are linked to the magnitude of cytotoxic T cell responses in human lung cancer. Nat Immunol.

[61] Koh J, Kim S, Kim MY, Go H, Jeon YK, Chung DH (2017). Prognostic implications of intratumoral CD103^+^ tumor-infiltrating lymphocytes in pulmonary squamous cell carcinoma. Oncotarget..

[62] Gu Y, Chen Y, Jin K, Cao Y, Liu X, Lv K, et al. Intratumoral CD103^+^ CD4^+^ T cell infiltration defines immunoevasive contexture and poor clinical outcomes in gastric cancer patients. 2020;9:e1844402-1-8. 10.1080/2162402X.2020.1844402 PMC7714530 33312758

[63] Lai C, Coltart G, Shapanis A, Healy C, Alabdulkareem A, Selvendran S, et al. CD8^+^CD103+ tissue-resident memory T cells convey reduced protective immunity in cutaneous squamous cell carcinoma. J Immunother Cancer. 2021;9:e001807. 10.1136/jitc-2020-001807 PMC7825273 33479027

[64] Sanders C, Salah A, Hamad M, Ng S, Hosni R, Ellinger J, et al. CD103^+^ tissue resident T-lymphocytes accumulate in lung metastases and are correlated with poor prognosis in ccRCC. Cancers. 2022;14:1541. 10.3390/cancers14061541 PMC8946052 35326691

[65] Duhen T, Duhen R, Montler R, Moses J, Moudgil T, De Miranda NF, et al. Co-expression of CD39 and CD103 identifies tumor-reactive CD8 T cells in human solid tumors. Nat Commun. 2018;9:1–13. 10.1038/s41467-018-05072-0 PMC6045647 30006565

[66] Timperi E, Barnaba V. Molecular sciences CD39 regulation and functions in T cells. Int J Mol Sci. 2021;22:7068. 10.3390/ijms22158068 PMC8348030 34360833

[67] Liu T, Tan J, Wu M, Fan W, Wei J, Zhu B (2021). High-affinity neoantigens correlate with better prognosis and trigger potent antihepatocellular carcinoma (HCC) activity by activating CD39^+^ CD8^+^ T cells. Gut..

[68] Zhang B, Cheng B, Li FS, Ding JH, Feng YY, Zhuo GZ (2015). High expression of CD39/ENTPD1 in malignant epithelial cells of human rectal adenocarcinoma. Tumor Biol.

[69] Wu J, Wang YC, Xu WH, Luo WJ, Wan FN, Zhang HL (2020). High expression of cd39 is associated with poor prognosis and immune infiltrates in clear cell renal cell carcinoma. Onco Targets Ther.

[70] Zhu W, Zhao Z, Feng B, Yu W, Li J, Guo H, et al. CD8+CD39+ T cells mediate anti-tumor cytotoxicity in bladder cancer. OncoTarget Ther. 2021;14:2149. 10.2147/OTT.S297272 PMC8006912 33790578

[71] Chen S, Wu S, Zhang L, Zhang W, Liu Y, Chen B (2020). CD39: the potential target in small cell lung cancer. Transl Lung Cancer Res.

[72] Giatromanolaki A, Kouroupi M, Pouliliou S, Mitrakas A, Hasan F, Pappa A (2020). Ectonucleotidase CD73 and CD39 expression in non-small cell lung cancer relates to hypoxia and immunosuppressive pathways. Life Sci.

[73] Han Y, Liu D, Li L (2020). PD-1/PD-L1 pathway: current researches in cancer. Am J Cancer Res.

[74] Lu JC, Zeng HY, Sun QM, Meng QN, Huang XY, Zhang PF (2019). Distinct PD-L1/PD1 profiles and clinical implications in intrahepatic cholangiocarcinoma patients with different risk factors. Theranostics.

[75] Webb JR, Milne K, Nelson BH (2015). PD-1 and CD103 are widely coexpressed on prognostically favorable intraepithelial CD8 T cells in human ovarian cancer. Cancer Immunol Res.

[76] Giatromanolaki A, Koukourakis IM, Konstantina B, Mitrakas AG, Harris AL, Michael (2019). Programmed death-1 receptor (PD-1) and PD-ligand-1 (PD-L1) expression in non-small cell lung cancer and the immune-suppressive effect of anaerobic glycolysis. Med Oncol.

[77] Thommen DS, Koelzer VH, Herzig P, Roller A, Trefny M, Dimeloe S (2018). A transcriptionally and functionally distinct PD-1^+^ CD8^+^ T cell pool with predictive potential in non-small-cell lung cancer treated with PD-1 blockade. Nat Med.

[78] Hummelink K, van der Noort V, Muller M, Schouten RD, Lalezari F, Peters D, et al. PD-1T TILs as a predictive biomarker for clinical benefit to PD-1 blockade in patients with advanced NSCLC. Clin Cancer Res. 2022;OF1–14. Available from: https://pubmed.ncbi.nlm.nih.gov/35852792/10.1158/1078-0432.CCR-22-0992 PMC9762332 35852792

[79] Li H, Yang D, Hao M, Liu H. Differential expression of HAVCR2 gene in pan-cancer: a potential biomarker for survival and immunotherapy. Front Genet. 2022;13:972664. 10.3389/fgene.2022.972664 PMC9445440 36081997

[80] Acar E, Esenda G, Yazıcı O (2022). Tumor-infiltrating lymphocytes (TIL), tertiary lymphoid structures (TLS), and expression of PD-1, TIM-3, LAG-3 on TIL in invasive and in situ ductal breast carcinomas and their relationship with prognostic factors. Clin Breast Cancer.

[81] Chen H, Molberg K, Carrick K, Niu S, Rivera Colon G, Gwin K, et al. Prevalence and prognostic significance of PD-L1, TIM-3 and B7-H3 expression in endometrial serous carcinoma. Mod Pathol. 2022. Available from: https://pubmed.ncbi.nlm.nih.gov/35804040/10.1038/s41379-022-01131-6 35804040

[82] Song D, Zhou Z, Wu J, Wei T, Zhao G, Ren H, et al. DNA methylation regulators-related molecular patterns and tumor immune landscape in hepatocellular carcinoma. Front Oncol. 2022;12:877817. 10.3389/fonc.2022.877817 PMC9459088 36091162

[83] Xiao K, Xiao K, Li K, Xue P, Zhu S. Prognostic role of TIGIT expression in patients with solid tumors: a meta-analysis. J Immunol Res. 2021;2021:5440572. 10.1155/2021/5440572 PMC8651431 34888386

[84] Saltz J, Gupta R, Hou L, Kurc T, Singh P, Nguyen V (2018). Spatial organization and molecular correlation of tumor-infiltrating lymphocytes using deep learning on pathology images. Cell Rep..

[85] Koelzer VH, Sirinukunwattana K, Rittscher J, Mertz KD. Precision immunoprofiling by image analysis and artificial intelligence. Virchows Archiv. 2019;474:511–522. 10.1007/s00428-018-2485-z PMC6447694 30470933

[86] Mobadersany P, Yousefi S, Amgad M, Gutman DA, Barnholtz-Sloan JS, Velázquez Vega JE, et al. Predicting cancer outcomes from histology and genomics using convolutional networks. Proc Natl Acad Sci USA. 2018;115:E2970–9. 10.1073/pnas.1717139115 PMC5879673 29531073

[87] Krizhevsky A, Sutskever I, Hinton GE. ImageNet classification with deep convolutional neural networks. 2022. Available from: http://code.google.com/p/cuda-convnet/

[88] Heindl A, Sestak I, Naidoo K, Cuzick J, Dowsett M, Yuan Y. Relevance of spatial heterogeneity of immune infiltration for predicting risk of recurrence after endocrine therapy of ER1 breast cancer. 2017. Available from: https://academic.oup.com/jnci/article-abstract/110/2/166/4064177 10.1093/jnci/djx137 PMC6298573 28859291

[89] Horeweg N, de Bruyn M, Nout RA, Stelloo E, Kedziersza K, Leon-Castillo A (2020). Prognostic integrated image-based immune and molecular profiling in early-stage endometrial cancer. Cancer Immunol Res.

[90] Tsakiroglou AM, Fergie M, Oguejiofor K, Linton K, Thomson D, Stern PL (2020). Spatial proximity between T and PD-L1 expressing cells as a prognostic biomarker for oropharyngeal squamous cell carcinoma. Br J Cancer.

[91] Chen Y, Sun Z, Chen W, Liu C, Chai R, Ding J, et al. The immune subtypes and landscape of gastric cancer and to predict based on the whole-slide images using deep learning. Front Immunol. 2021;12:685992. 10.3389/fimmu.2021.685992 PMC8273735 34262565

[92] Duruisseaux M, Martínez-Cardús A, Calleja-Cervantes ME, Moran S, Castro de Moura M, Davalos V (2018). Epigenetic prediction of response to anti-PD-1 treatment in non-small-cell lung cancer: a multicentre, retrospective analysis. Lancet Respir Med.

[93] Yamashita R, Long J, Longacre T, Peng L, Berry G, Martin B (2021). Deep learning model for the prediction of microsatellite instability in colorectal cancer: a diagnostic study. Lancet Oncol..

[94] Schrammen PL, Ghaffari Laleh N, Echle A, Truhn D, Schulz V, Brinker TJ (2022). Weakly supervised annotation-free cancer detection and prediction of genotype in routine histopathology. J Pathol.

[95] Bilal M, Raza SEA, Azam A, Graham S, Ilyas M, Cree IA (2021). Development and validation of a weakly supervised deep learning framework to predict the status of molecular pathways and key mutations in colorectal cancer from routine histology images: a retrospective study. Lancet Digit Health.

[96] Lee SH, Song IH, Jang HJ (2021). Feasibility of deep learning-based fully automated classification of microsatellite instability in tissue slides of colorectal cancer. Int J Cancer.

[97] Kather JN, Pearson AT, Halama N, Jäger D, Krause J, Loosen SH (2019). Deep learning can predict microsatellite instability directly from histology in gastrointestinal cancer. Nat Med.

[98] Presti D, Dall’Olio FG, Besse B, Ribeiro JM, Di Meglio A, Soldato D. Tumor infiltrating lymphocytes (TILs) as a predictive biomarker of response to checkpoint blockers in solid tumors: a systematic review. Crit Rev Oncol Hematol. 2022;177:103773. 10.1016/j.critrevonc.2022.103773 35917885

[99] Bianchini G, Huang CS, Egle D, Bermejo B, Zamagni C, Thill M (2020). LBA13 tumour infiltrating lymphocytes (TILs), PD-L1 expression and their dynamics in the NeoTRIPaPDL1 trial. Ann Oncol.

[100] Tumeh PC, Harview CL, Yearley JH, Shintaku IP, Taylor EJM, Robert L (2014). PD-1 blockade induces responses by inhibiting adaptive immune resistance. Nature.

[101] Gide TN, Silva IP, Quek C, Ahmed T, Menzies AM, Carlino MS, et al. Close proximity of immune and tumor cells underlies response to anti-PD-1 based therapies in metastatic melanoma patients. Oncoimmunology. 2019;9:1659093. 10.1080/2162402X.2019.1659093 PMC6959449 32002281

[102] Harder N, Schönmeyer R, Nekolla K, Meier A, Brieu N, Vanegas C (2019). Automatic discovery of image-based signatures for ipilimumab response prediction in malignant melanoma. Sci Rep..

[103] Madonna G, Ballesteros-Merino C, Feng Z, Bifulco C, Capone M, Giannarelli D, et al. PD-L1 expression with immune-infiltrate evaluation and outcome prediction in melanoma patients treated with ipilimumab. Oncoimmunology. 2018;7:e1405206. 10.1080/2162402X.2017.1405206 PMC6279420 30524879

[104] Wong PF, Wei W, Smithy JW, Acs B, Toki MI, Blenman KRM (2019). Multiplex quantitative analysis of tumor-infiltrating lymphocytes and immunotherapy outcome in metastatic melanoma. Clin Cancer Res.

[105] Loupakis F, Depetris I, Biason P, Intini R, Prete AA, Leone F (2020). Prediction of benefit from checkpoint inhibitors in mismatch repair deficient metastatic colorectal cancer: role of tumor infiltrating lymphocytes. Oncologist.

[106] Kato K, Doki Y, Ura T, Hamamoto Y, Kojima T, Tsushima T (2020). Long-term efficacy and predictive correlates of response to nivolumab in Japanese patients with esophageal cancer. Cancer Sci.

[107] Konstantinopoulos PA, Luo W, Liu JF, Doga, Gulhan C, Krasner C (2019). Phase II study of avelumab in patients with mismatch repair deficient and mismatch repair proficient recurrent/persistent endometrial cancer. J Clin Oncol.

[108] Pignon JC, Jegede O, Shukla SA, Braun DA, Horak CE, Wind-Rotolo M (2019). Irrecist for the evaluation of candidate biomarkers of response to nivolumab in metastatic clear cell renal cell carcinoma: analysis of a phase II prospective clinical trial. Clin Cancer Res.

[109] Ficial M, Jegede OA, Angelo MS, Hou Y, Flaifel A, Pignon JC (2021). Expression of T-Cell exhaustion molecules and human endogenous retroviruses as predictive biomarkers for response to nivolumab in metastatic clear cell renal cell carcinoma. Clin Cancer Res.

[110] Zheng L, Qin S, Si W, Wang A, Xing B, Gao R, et al. Pan-cancer single-cell landscape of tumor-infiltrating T cells. Science. 2021;374:abe6474. 10.1126/science.abe6474 34914499

[111] Gataa I, Mezquita L, Rossoni C, Auclin E, Kossai M, Aboubakar F (2021). Tumour-infiltrating lymphocyte density is associated with favourable outcome in patients with advanced non–small cell lung cancer treated with immunotherapy. Eur J Cancer.

[112] Amaria RN, Reddy SM, Tawbi HA, Davies MA, Ross MI, Glitza IC (2018). Neoadjuvant immune checkpoint blockade in high-risk resectable melanoma. Nat Med.

[113] Uryvaev A, Passhak M, Hershkovits D, Sabo E, Bar-Sela G. The role of tumor-infiltrating lymphocytes (TILs) as a predictive biomarker of response to anti-PD1 therapy in patients with metastatic non-small cell lung cancer or metastatic melanoma. Med Oncol. 2018;35:1–9. 10.1007/s12032-018-1080-0 29388007

[114] Spassova I, Ugurel S, Terheyden P, Sucker A, Hassel JC, Ritter C (2020). Predominance of central memory T cells with high T-cell receptor repertoire diversity is associated with response to PD-1/PD-L1 inhibition in Merkel cell carcinoma. Clin Cancer Res.

[115] Schoenfeld JD, Hanna GJ, Jo VY, Rawal B, Chen YH, Catalano PS (2020). Neoadjuvant nivolumab or nivolumab plus ipilimumab in untreated oral cavity squamous cell carcinoma: a phase 2 open-label randomized clinical trial. JAMA Oncol.

[116] Loeffler DA, Juneau PL, Masserant S. Influence of tumour physico-chemical conditions on interleukin-2-stimulated lymphocyte proliferation. Br J Cancer. 1992;66:619–22. 10.1038/bjc.1992.326 PMC1977437 1419598

[117] Gaggero S, Martinez-Fabregas J, Cozzani A, Fyfe PK, Leprohon M, Yang J, et al. IL-2 is inactivated by the acidic pH environment of tumors enabling engineering of a pH-selective mutein. Sci Immunol. 2022;7:eade5686. 10.1126/sciimmunol.ade5686 36459543

[118] Calcinotto A, Filipazzi P, Grioni M, Iero M, de Milito A, Ricupito A (2012). Modulation of microenvironment acidity reverses anergy in human and murine tumor-infiltrating T lymphocytes. Cancer Res.

[119] Multhoff G, Vaupel P (2020). Hypoxia compromises anti-cancer immune responses. Adv Exp Med Biol.

[120] Zandberg DP, Menk AV, Velez M, Normolle D, Depeaux K, Liu A, et al. Tumor hypoxia is associated with resistance to PD-1 blockade in squamous cell carcinoma of the head and neck. J Immunother Cancer. 2021;9:e002088. 10.1136/jitc-2020-002088 PMC8126285 33986123

[121] Peng H, He X, Wang Q (2022). Immune checkpoint blockades in gynecological cancers: a review of clinical trials. Acta Obstet Gynecol Scand.

[122] Khatoon E, Parama D, Kumar A, Alqahtani MS, Abbas M, Girisa S (2022). Targeting PD-1/PD-L1 axis as new horizon for ovarian cancer therapy. Life Sci.

